# Contribution of Step Length to Increase Walking and Turning Speed as a Marker of Parkinson’s Disease Progression

**DOI:** 10.1371/journal.pone.0152469

**Published:** 2016-04-25

**Authors:** Nicolas Bayle, Amar S. Patel, Diana Crisan, Lanjun J. Guo, Emilie Hutin, Donald J. Weisz, Steven T. Moore, Jean-Michel Gracies

**Affiliations:** 1 Laboratoire *Analyse et Restauration du Mouvement*, EA BIOTN 7377, Hôpitaux Universitaires Henri Mondor, AP-HP, Université Paris-Est Créteil, Créteil, France; 2 Robert and John M. Bendheim Parkinson and Movement Disorders Center, Department of Neurology, Mount Sinai School of Medicine, New York, United States of America; 3 Neurosurgery Department, Mount Sinai School of Medicine, New York, NY, United States of America; 4 Human Aerospace Laboratory, Department of Neurology, Mount Sinai School of Medicine, New York, United States of America; Cardiff University, UNITED KINGDOM

## Abstract

When increasing ambulation speed in Parkinson’s disease, step cadence increases more than stride length, indicating movement scaling difficulties that affect step generation in particular. We investigated whether step length variation when increasing ambulation speed was related to disease progression. Patients with Parkinson’s disease (N = 39) and controls (N = 152) performed two timed ambulation tasks: at a 'free' (self-selected) pace and then at 'maximal' speed. The total number of steps (including during turns) and time to complete the task were clinically measured. The relative contribution of step length and cadence to increased ambulation speed was determined using two methods: the ratios of change in step length or in cadence to the change in ambulation speed, and the step length index. While the relative contribution of step length and cadence to increased ambulation speed was independent of age in both control and patient groups, in Parkinson’s disease there was a negative correlation between time from diagnosis and the ratio of change in step length to change in ambulation speed (R = 0.54; *p* = 0.0004) and the step length index (R = 0.56, *p* = 0.0002). In parallel, there was a positive correlation between time since diagnosis and the ratio of change in cadence to change in ambulation speed (R = 0.57; *p* = 0.0002). The relative contribution of step length and cadence to increased ambulation speed is age invariant but a marker of Parkinson's disease advancement, and can be easily determined in the clinical setting.

## Introduction

James Parkinson noted moderately advanced patients as “being, at the same time, irresistibly impelled to take much quicker and shorter steps, and thereby to adopt unwillingly a running pace”[1]. Slowness of gait, small and variable step length and festination have since been considered classic clinical features in Parkinson’s disease (PD) [2]. Laboratory studies have demonstrated that step length regulation is defective in PD while the ability to modulate ambulation cadence is relatively preserved [3–5]. Specifically, Morris and colleagues have previously suggested that in Parkinson’s disease, step cadence increased as stride length decreased while walking from slow to medium walking speeds, indicating that the movement scaling difficulty of the disease particularly affects step production [5]. However, classic clinical examination of Parkinson’s disease (UPDRS III) does not specifically measure the movement scaling difficulty of the disease; furthermore, a number of motor features of PD (abnormal posture, bradykinesia, decreased step length) also occur with normal aging [6–8]. The search for quantitative markers of disease progression that are independent of age is important, both to enhance our basic understanding of PD and to facilitate the investigation of new potential neuroprotective therapies [9].

As far as ambulation is concerned, movement scaling difficulties lead to utilize more steps to cover a given distance in PD, a concept we can refer to as *step consumption*. A step consumption increase will be reflected by both an increase in cadence and a decrease in step length. To investigate abnormal step consumption as a possible marker of disease progression, we evaluated PD patients at various ages and disease durations in comparison to a large cohort of healthy individuals across various age groups. We hypothesized that an excessive step consumption increase with speed increase when ambulating faster in PD, which would be a marker of the disease and prove of value as a simple quantitative index of disease progression in the clinical setting. The clinical relevance of such finding would be to propose a simple test (quantification of the number of steps and seconds to walk normally and then fast over a specific distance) in the armamentarium of clinicians involved with PD patients, so they could derive a quantitative index as to the progression of the disease. Finally, we explored the effects of levodopa on step consumption, hypothesizing that levodopa may reduce step consumption by increasing ability to increase step length upon ambulation speed increase [10,11].

## Methods

### PD patients

This patient study was a retrospective chart review. The experimental procedure was in accordance with the declaration of Helsinski (2008) and approved by the Mount Sinai School of Medicine Institutional Review Board (IRB). All healthy subjects and PD patients gave a written informed consent. Thirty nine consecutive patients with Parkinson’s disease based on the United Kingdom-Parkinson’s Disease Society Brain Bank (UK-PDSBB) [12] criteria (29M, 10F, age 64 ± 7 [48–78]; disease duration 9 ± 6; Hoehn and Yahr Stage 2–4; mean UPDRS OFF 31.4 ± 10.1; UPDRS ON 21.5 ± 10.6; levodopa-equivalent daily dose, LEDD, 700.4 ± 495 mg/day; Table 1) were routinely evaluated for ambulation at the Mount Sinai Movement Disorders Clinic using two ambulation tasks (modified Timed up-and-go) as described below. Following classical guidelines, the first assessment was performed in the clinically defined OFF-status after drug withdrawal for at least 12 hours for levodopa and 48 hours for other antiparkinsonian drugs (“med off”) [13,14]. We also observed PD patients in the ON state, approximately one hour after taking their regular levodopa dose. Performances in the OFF and ON states were both analyzed.

**Table 1:** Subject characteristics.

	Healthy	Healthy Age-matched	PD
**Number**	152	39	39
**Age (years)**	45 (14)	63 (5)	64(7)
**Gender**	76F	10F	10F
**Time since diagnosis (years)**	_____________	_____________	9 (6)
**Hoehn and Yahr**	_____________	_____________	2–4
**Mea, UPDRS off/on**	_____________	_____________	31.4±10.1/21.5±10.6
**LEDD(mg/day)**			700.4 ± 494.9

### Control subjects

We recruited 152 healthy subjects (76M, 76F, age 45±14 [19–78]), with no history of neurological disorders or gait abnormalities, to participate in a research study on human movement. Each subject signed an informed consent approved by the Mount Sinai School of Medicine IRB. Healthy subjects were asked to perform the ambulation task described above, as performed by PD patients during clinic visits. Out of this group, we selected an age- and sex-matched control group of the 39 oldest subjects (29M, 10F, age 63 ± 5 [57–78]); the 113 remaining subjects constituted a 'healthy young' group (47M, 66F, age 39 ± 10 [20–57]).

### Assessments

**Fig 1:**
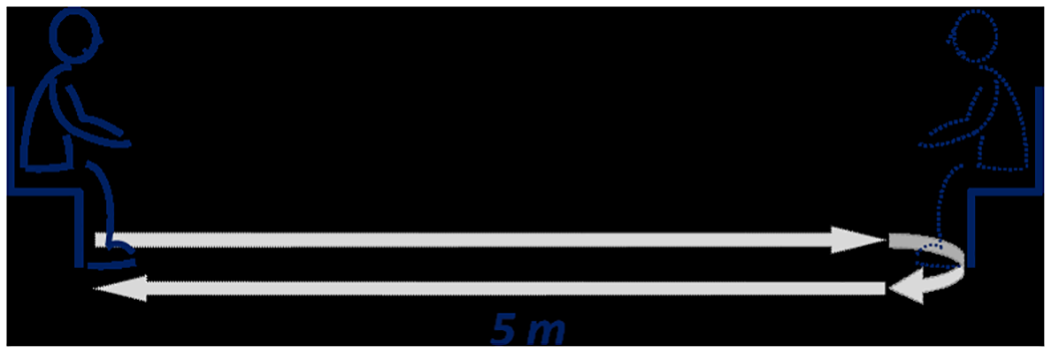
Ambulation task. each trial started with subjects in the sitting position in a first chair, from which they stood up, walked 5 m to a second chair, turned 180° before sitting in the second chair, stood up again, walked 5 m back to the starting point, turned 180° and then sat down in the first chair.

To both better reflect real life conditions and sensitize the exploration of step consumption, we studied ambulation rather than straight gait only, and specifically measured step consumption changes when ambulating (walking, turning) at a self-selected (free) pace and at a maximal safe (fast) speed. All trials were identical for all participants, including for the healthy subjects (section below). The ambulation task used here can be considered as a modified, more complete version of the standard Timed Up-and-Go test [15]: each trial started with subjects in the sitting position in a first chair, from which they stood up, walked 5 m to a second chair, turned 180° before sitting in the second chair, stood up again, walked 5 m back to the starting point, turned 180° and then sat down in the first chair (Fig 1). The first trial was performed at a free pace, and second at safe fast speed. Time of completion was measured using a stop watch, triggered from onset of sit-to-stand (‘backside off’) to end of stand-to-sit (‘backside on’). The total step consumption was measured, by counting all the steps taken to walk *and* for each u-turn preceding each stand-to-sit. Thus, the methodology of the test did not require to separate straight line walking from turning since *all* steps were counted, from the first sit-to-stand to the last stand-to-sit, including u-turns. Any freezing episode was not specifically inventoried. The clinician only counted each step used for the whole ambulation task, i.e. each full foot take off, whether occurring during freezing episodes or not.

### Ambulation parameters

From here on, the parameter *step length* is defined as the total number of meters covered in the test (10 meters) divided by the total number of steps, counted as described above (the “angular” displacement covered during each u-turn was not counted). We thus determined mean ambulation characteristics, step length (SL), cadence (CAD, also termed step frequency), and ambulation speed (SP), in the control and patient groups from the time and the number of steps counted as specified above at the self-selected and fast speeds. We calculated the percent increase in mean ambulation parameters from free to fast ambulation as:

$$\Delta SL=\frac{SL_{fast}-SL_{free}}{SL_{free}}*100,$$

$$\Delta CAD=\frac{CAD_{fast}-CAD_{free}}{CAD_{free}}*100,$$

$$\Delta SP=\frac{SP_{fast}-SP_{free}}{SP_{free}}*100.$$

The step length index (SLI), a measure of the percent change in speed due to changes in step length, has previously been defined [16]. SLI compares the logarithm of the ratio of the two different step lengths to the logarithm of the ratio of the two different ambulation velocities, computed as follows:

$$SLI=\frac{\text{log}(SL_{fast}/SL_{free})}{\text{log}(SP_{fast}/SP_{free})}*100.$$

Since walking velocity is equally dependent on cadence and step length,

SP=SL*CAD

a SLI of 50% implies equal contributions of step length and cadence for a given change in ambulation speed. An SLI of 0% signified that a change of ambulation velocity was attributed totally to a change in cadence; an SLI of 100% indicated that a change of step length was solely responsible; uncommon cases of SLI over 100% would indicate that a major increase in step length compensated for a cadence reduction in the fast speed ambulation task; uncommon cases of SLI below 0% would indicate a reduction of step length, which would be compensated by a major cadence increase in the fast speed ambulation task [16]. We performed regression analyses to determine the relationship between the step length index and age in healthy subjects and PD patients, and between the step length index and time since diagnosis in PD patients.

We also defined two simpler ratios with the aim of providing more practical parameters for use in a clinical setting: the ratio of the increase in mean step length over the increase in mean ambulation speed (ΔSL/ΔSP) as an estimation of the contribution of step length (CSL) increase to speed increase, and the ratio of the increase in mean cadence over the increase in mean ambulation speed (ΔCAD/ΔSP) as an estimation of the contribution of cadence (CCAD) increase to speed increase. Regression analyses examined the relationship of CSL and CCAD to SLI. We then evaluated age and time since diagnosis as predictors of SLI, CSL and CCAD.

### Statistics

Descriptive statistics were performed to calculate the average values and standard deviations for each of the variables considered (age, time since diagnosis, UPDRS III, levodopa-equivalent daily dose, step length, cadence, ambulation speed, step length increase, cadence increase, ambulation speed increase, and contributions of step length and cadence to ambulation speed) in both PD and healthy cohorts. One-way analysis of variance (ANOVA) was used to compare the groups of PD patients (in the OFF state), age-matched and young controls. Univariable linear regression analysis was performed to evaluate the importance of age in healthy subjects–and of age, time since diagnosis, UPDRS III and levodopa-equivalent daily dose (LEDD) in PD patients–as potential predictors of step length, cadence, ambulation speed, and contributions of step length and cadence increases to ambulation acceleration. In a second stage, multivariable analysis was performed in the PD group adjusting for age, time since diagnosis, UPDRS III and levodopa-equivalent dose (LED) as independent predictor variables. The significance of linear relationships was evaluated with an F-test (ANOVA). Paired *t*-tests were used to compare PD patients in the OFF and in the ON states. Bonferroni corrections were used to account for multiple comparisons. The data were analyzed using SPSS 17.0 for Windows (Chicago, IL.). A significance level of p < 0.05 was used.

## Results

### Healthy subjects

**Fig 2:**
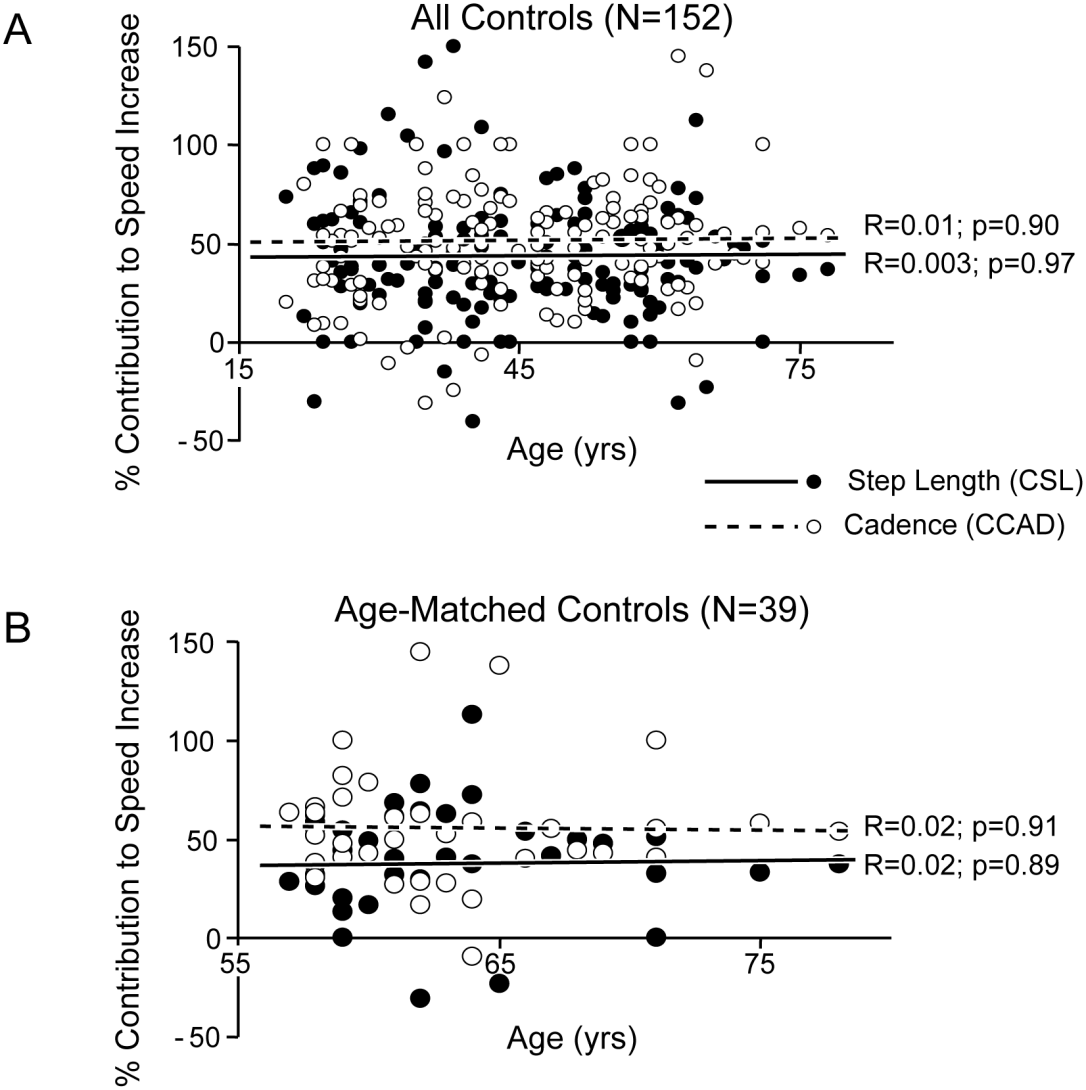
Age and contributions of step length and cadence change to increased ambulation speed. Contributions of step length (CSL, ΔSL/ΔSP) and cadence (CCAD, ΔCAD/ΔSP) to speed (SP) increase, as a function of age in all controls (A) and age-matched controls (B).

**Fig 3:**
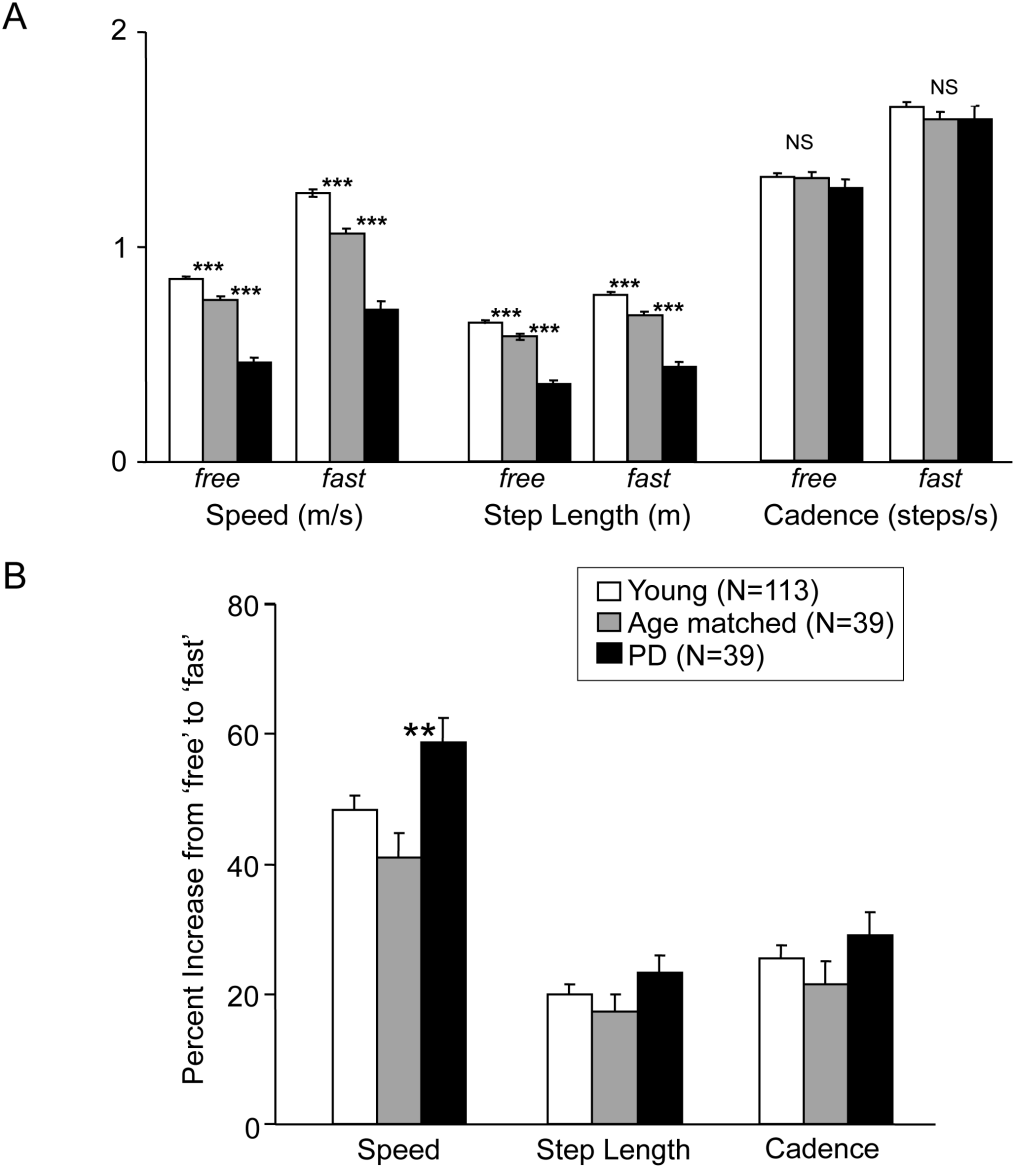
Mean ambulation characteristics in all subjects groups. Results expressed in mean±SEM, as absolute values of speed, step length and cadence (A), and percent change in parameters from free to fast speed (B). ***, *p* < 0.001, **, *p* < 0.01 (pairwise comparisons, ANOVA).

Advancing age was associated with a reduction in both ambulation speed and step length, particularly at maximal safe speed, while there was only a trend for cadence reduction (Table 2A). The increase in speed from free to maximal safe speed (46 ± 16%) also diminished with age (R = 0.19, *p* = 0.02); however, age was not a predictor of the variations of step length increase (R = 0.05, *p* = 0.52) or of cadence increase (R = 0.11, *p* = 0.19) in this sample (Table 2B). The mean step length index (SLI) was 45 ± 31%. SLI did not correlate with age in either the healthy young (R = 0.07, *p* = 0.47) or age-matched groups (R = 0.02, *p* = 0.90; data not shown). The estimated contribution of step length increase to ambulation acceleration (CSL) was 42 ± 30% on average, closely correlated with SLI (R = 0.99; *p* < 0.001; data not shown) and also not correlated with age (R = 0.003, *p* = 0.97; Fig 2A), even in the age-matched group (Fig 2B). Thus, as a group, the age-matched subjects walked with a preserved cadence but smaller step length and speed than the young healthy subjects, both at free and maximal safe ambulation speed (*p* < 0.001, one-way ANOVA; Fig 3A). However, their mean increase in speed, step length, and cadence was not significantly different from that of the young healthy subjects (Fig 3B).

**Table 2:** Ambulation parameters *vs* age and time since diagnosis. Correlation between age or time since diagnosis and ambulation parameters at free and fast speed (A); parameter changes from free to fast speed (B). R, correlation coefficient; Δ, change from free to fast speed.

	*vs*. *Age*		*vs*. *Time since Diagnosis*	
	R	p	R	p
**2A Ambulations**				
**Parameters**				
***Healthy Subjects***				
Speed free	0,45	<0,0001	NA	NA
Speed fast	0,56	<0,0001	NA	NA
Step length free	0,38	<0,0001	NA	NA
Step length fast	0,34	<0,0001	NA	NA
Cadence free	0,07	0,34	NA	NA
Cadence Fast	0,15	0,06	NA	NA
***PD Subjects***				
Speed free	0,19	0,26	0,49	0,001
Speed fast	0,31	0,57	0,57	<0,0001
Step length free	0,04	0,81	0,59	<0,0001
Step length fast	0,13	0,44	0,63	<0,0001
Cadence free	0,26	0,11	0,37	0,02
Cadence Fast	0,3	0,06	0,18	0,28
**2B Changes adjusted for values at free speed**				
***Healthy Subjects***				
ΔSpeed	0,19	0,02	NA	NA
ΔStep Length	0,05	0,52	NA	NA
ΔCadence	0,11	0,19	NA	NA
***Young Healthy Subjects***				
ΔSpeed	0,54	0,02	NA	NA
ΔStep Length	0,1	0,39	NA	NA
ΔCadence	0,32	0,77	NA	NA
***Age-Matched Helathy Subjects***				
ΔSpeed	0,47	0,4	NA	NA
ΔStep Length	0,05	0,93	NA	NA
ΔCadence	0,3	0,59	NA	NA
***PD Subjects***				
ΔSpeed	0,5	0,24	0,47	0,67
ΔStep Length	0,42	0,32	0,57	0,006
ΔCadence	0,44	0,49	0,58	0,008

### PD patients

In PD subjects, step length and speed were significantly less than those of both healthy groups at free and fast pace (*p* = 0.001, one-way ANOVA; Fig 3A) but cadence was on average the same as in healthy subjects (whole population and age-matched subgroup; Fig 3A). There was a greater increase in speed from free to maximal safe speed in PD patients than in controls (Fig 3B). Ambulation speed and step length significantly decreased with time from diagnosis but not with age (Table 1A). As with healthy subjects, step length and cadence increase did not vary with age when adjusting for values at free ambulation speed in bivariable analysis. In contrast, time from diagnosis was a strong predictor of both variables (ΔSL, R = 0.57, *p* = 0.006; ΔCAD, R = 0.58, *p* = 0.008; Table 1B).

**Fig 4:**
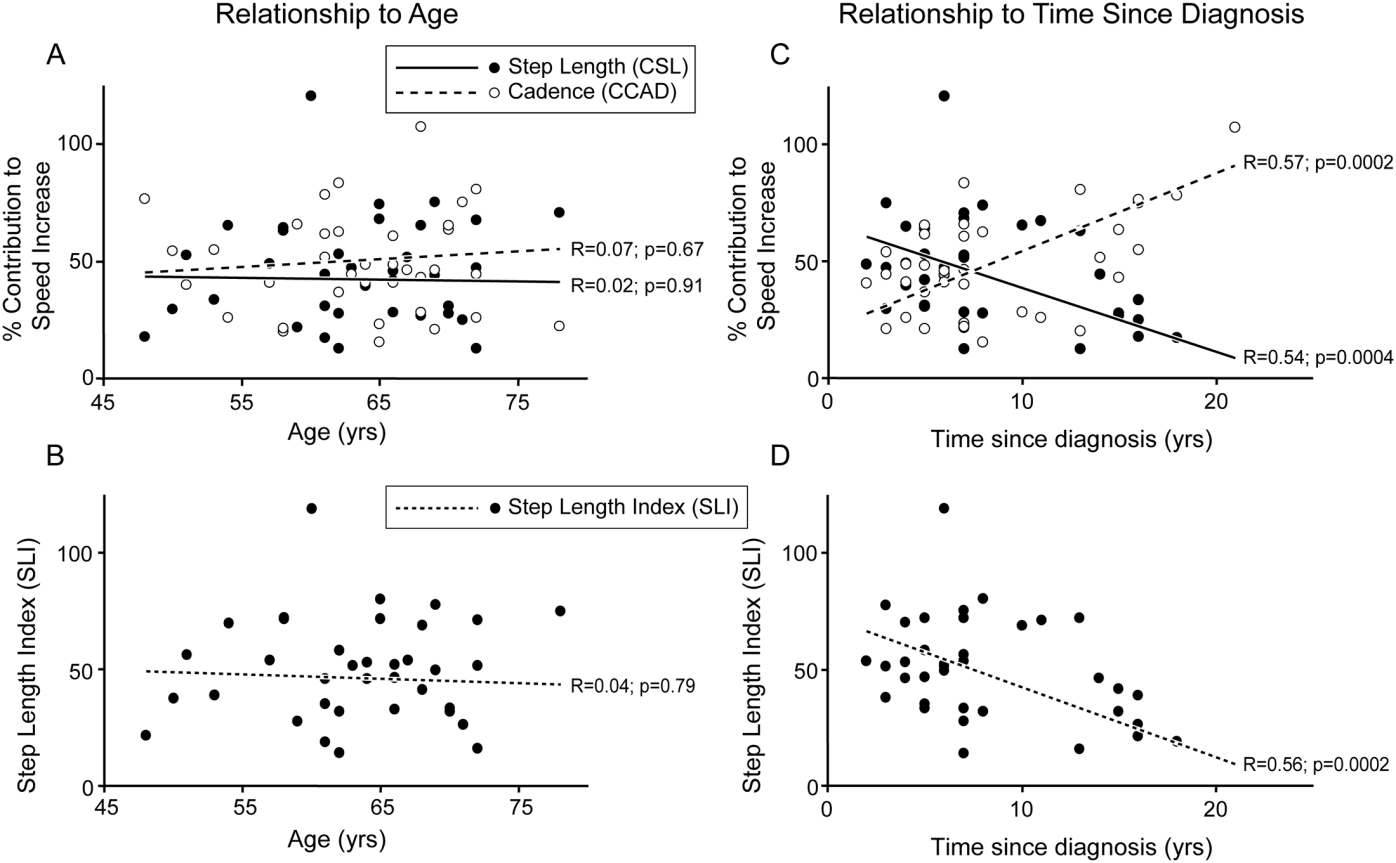
Contributions of step length and cadence change to increased ambulation speed *vs* age or time since diagnosis in patients with PD. Contributions of step length (CSL) and cadence (CCAD) to speed increase as a function of age (A) and time since diagnosis (B). Step length Index (SLI) as a function of age (C), and time since diagnosis (D).

The mean step length index (SLI) was 46 ± 28%, which was not different from healthy subjects. SLI did not vary with age (R = 0.04, *p* = 0.79) but decreased significantly with time from diagnosis (R = 0.56, *p* = 0.0002; Fig 4D). The mean SLI for up to 8 years from time of diagnosis was 54 ± 22%, but fell to 31 ± 33% after 8 years since diagnosis (*p* < 0.05, *t*-test).

The estimated contribution of step length (CSL) to speed increase was 42 ± 26% on average in PD, which was not different from healthy subjects. CSL was closely correlated with SLI (R = 0.99; *p* < 0.001; data not shown) and also did not vary with age (Fig 4A). The mean CSL for up to 8 years from time of diagnosis was 50 ± 22% but was only 28 ± 28% after 8 years since diagnosis (*p* < 0.05, *t*-test). Like the SLI, the CSL also significantly diminished with time from diagnosis in univariable analysis (R = 0.54; *p* = 0.0004; Fig 4C), while the contribution of cadence (CCAD) reciprocally and significantly increased with time from diagnosis (R = 0.58; *p* = 0.0002; Fig 4C). Such relations were not observed with UPDRS III (R = 0.02; *p* = 0.99) or LEDD (R = 0.038; *p* = 0.86; data not shown). These regressions persisted when adjusting for age, UPDRS III or LEDD in a multivariable regression analysis, as time from diagnosis remained a highly significant negative predictor of CSL (R = 0.66; slope ß = -0.029; *p* < 0.0001) and a positive predictor of CCAD (R = 0.57; slope ß = 0.033; *p* = 0.0002), while age (CSL, slope ß = 0.003; *p* = 0.63; CCAD, slope ß = 0.001; *p* = 0.94), UPDRS III (CSL, slope ß = 0.32; *p* = 0.09) or LEDD (CSL, slope ß = 0.23; *p* = 0.22) were not predictors. Further adjustments for step length or cadence at free speed, or their changes between free and fast speed, were not useful because these variables are all linearly related to time from diagnosis and therefore introduce collinearity in the model (Table 1B).

### Effects of levodopa

PD patients walked significantly faster on levodopa than off levodopa at both free speed (30% increase; OFF, 0.47 ± 0.16 m/s; ON, 0.61 ± 0.14 m/s, *p* = 1.4E-6, *t*-test) and maximal safe speed (15% increase; OFF, 0.72 ± 0.24 m/s; ON, 0.83 ± 0.17 m/s, *p* = 0.0020, *t*-test). The speed increase associated with the medication involved primarily an increase in step length, at both free speed (26% increase; OFF, 0.35 ± 0.10 m; ON, 0.44 ± 0.12 m, *p* = 8.2E-6, *t*-test) and maximal safe speed (23% increase; OFF, 0.44 ± 0.12 m; ON, 0.54 ± 0.10 m, *p* = 7.8E-6, *t*-test). The effect of levodopa on cadence was less marked; cadence at maximal safe speed was in fact non-significantly lower on levodopa than off levodopa (selected speed, OFF, 1.30 ± 0.26 step/s; ON, 1.37 ± 0.22 step/s, *p* = 0.032; maximal speed, OFF, 1.64 ± 0.37 step/s; ON, 1.55 ± 0.23 step/s, *p* = 0.2, *t*-test). There was a trend for a greater contribution of step length increase to ambulation acceleration (CSL) on levodopa (60.2 ± 50.9%) than off levodopa (44.6 ± 27.5%, *p* = 0.11).

## Discussion

This study demonstrates that as their disease advanced, patients increasingly relied on an increase in cadence to increase ambulation velocity. Thus, the *contribution of step length* (CSL) when increasing ambulation speed, expressed as a simple ratio of the change in step length to the change in ambulation speed during an ambulation task, was a marker of disease advancement in PD patients in clinical settings, as CSL did not change with age in healthy subjects or in PD. This parameter has good construct validity, correlating strongly with a published metric of step length contribution to walking speed increase (step length index) [16]. CSL is readily calculated from clinical measures during the two-speed ambulation task used (time to complete and number of steps).

### Limitations of the study

This was a retrospective study based on a chart review, as opposed to a prospective longitudinal study in which the same patients would have been followed over years. Therefore, caution must be exercised before really ascribing the gradual loss of movement scaling here to disease progression. We can only consider it to be a marker of disease duration. Admittedly, what is termed *step length* throughout this manuscript is a small underestimation of the step length that could be measured during straight gait since the (angular) displacement covered over two u-turns was not counted (thus the sum of the CSL and the CCAD did not amount exactly to 100%, see Fig 2). Similarly, speed measurements (distance divided by the number of seconds taken to complete the tasks, using a manually triggered stopwatch), were slightly underestimated, for the same reason. Cadence, which was the number of steps divided by the number of seconds, was not subject to the same underestimation. However, the underestimation of step length and speed was the same for each patient and in each walking condition and is likely to have been by a relatively constant factor (180° twice) in each case. Still, the regulation of step consumption in a simple timed ambulation test is shown to be abnormal in this study of Parkinson’s disease and to be potentially helpful as a marker of disease advancement.

Hypometria is the fundamental clinical characteristic of movement impairment in PD, and is manifested during ambulation tasks as step shortening [3–5]. However, it has been difficult to characterize Parkinson’s disease independently of aging, as the elderly population shares several key motor features with PD, such as abnormal posture, slower ambulation speed and smaller movement size in general [6–8]. The present study confirmed that ambulation speed and step length markedly decrease with age while cadence does not [3–5, 17, 18]. It also indicated that the contribution of step length increase (CSL) to ambulation velocity increases is age-invariant, in healthy controls as in PD. In contrast, we found that PD patients show a specific deficit in this ability, which becomes a consistent feature late in the disease evolution. However, since CSL becomes consistently abnormal only late in the disease (Fig 4C), this parameter may not be useful as a criterion for early diagnosis. To our knowledge, step consumption regulation, expressed as the ability to increase step length when accelerating ambulation, has not been explored as a function of age or time since diagnosis in PD.

In this retrospective study, the parameter considered was time since diagnosis and not time since the first manifestation of the disease. It is a fact that time since diagnosis might not be the ideal parameter to evaluate in this type of investigation, as this specific time may be dependent on the physician—and on access to specialized physicians—as much as on the patient history. However, we can assume, in the urban environment in which our study was conducted, that time since diagnosis would correlate strongly with time since the first manifestation of the disease. Outside a prospective protocol with a procedure defined a priori to ascertain the “first manifestations” of the disease, it might be difficult in retrospect for the investigator to ascertain whether some non-specific complaints (pain, depression, etc.) might have constituted the real onset of the disease process, or not, in an individual case. Finally, we based clinical diagnosis of Parkinson’s disease on the UK-PDSBB criteria, which may have allowed patients with atypical parkinsonism into our subject population, even though patients with atypical features were excluded and all diagnoses were confirmed by a movement disorders specialist [19].

### Medication effect

The relatively low ambulation speeds in patients with PD in the OFF and ON medication conditions in the present study, compared with gait speeds measured during simple walking tasks [20], may represent the difficulty with turning, standing up and sitting down seen in PD. The effect of levodopa on ambulation further emphasizes the importance of step length deficiencies as a characteristic of PD. Levodopa increased ambulation speed in PD patients at both free and maximal safe speed mainly through an increase in step length, which corroborates other reports [10, 11]. Also, there was a trend to enhance the contribution of step length increase to ambulation acceleration on levodopa.

### Pathophysiological considerations

The lacking ability to extend step length upon the demand of a faster gait corresponds to hypometria. Hypometria in PD has been explained by the inability to generate initial agonist bursts of sufficient duration or size and therefore by insufficient movement acceleration [21–28]. In Alexander’s model, this is due to inappropriate preparedness (low “internal” excitability) of the premotor and motor cortex by the basal ganglia [29]. The ability to make steps follow each other as fast as possible (step cadence increase) may be more dependent on set shifting ability [30]. It is possible that hypometria consistently worsens with disease duration in Parkinson’s disease while set shifting ability might not follow this pattern of change. The lack of correlation between UPDRS III scores and CSL may not be surprising as there are no items in UPDRS that specifically measure hypometria [31].

### Clinical use of the 10-meter ambulation test

An important characteristic of this study is the composite nature of the ambulation assessment used, which combined walking, turning, sit-to-stand and stand-to-sit tasks. We used this test to better reflect real life conditions, in which walking is never an isolated task, but also with the hypothesis that this would be a more sensitive clinical tool to reflect impairment in step consumption than straight gait alone. Less challenging but comparable timed up-and-go tests have shown good test-retest reliability and sensitivity to medication-induced changes [15]. Recent personal data shows the reliability of a longer, 20 m (twice 10 meters)-ambulation test in Parkinson’s disease. Combining these data with the test-retest reliability shown by Morris et al in the 6 meter (twice 3 meters) ambulation test makes it likely that the present 10-meter test (twice 5 meters) test has similar reliability. In terms of sensitivity to change and of validity as against other clinical measures in Parkinson’s disease, the present study shows that CSL over the 10-meter ambulation task is responsive to levodopa intake and correlates with disease duration but not with clinical UPDRS scores. In other words the test might detect a specific movement scaling difficulty in the lower limbs that may not be captured when performing conventional UPDRS testing. Another feature of this study is that we did not assess ambulation in the controlled environment of a gait laboratory or use objective ambulation analysis measures. However, walking speed measurements in the clinic setting have high inter-rater reliability when measured using a stopwatch and high concurrent validity with infrared timing gate measurement procedures [32]. In fact, one objective of the study was to demonstrate the feasibility and value of manual quantitative ambulation assessments in the clinical setting, in which complex motion capture analysis is often impractical.

The present study finally pointed out an interesting feature of Parkinson’s disease. The percent increase in speed from free to maximal safe ambulation speed was greater in PD patients (off levodopa) than in controls, in whom this percent increase even diminished with age (Fig 2B, Table 1B). In other words, the free, self-selected speed was slower in PD patients than in controls relative to maximal safe speed. Analogous findings have been reported when testing walking over longer distances [33,34]. This may refer to a behavior specific to PD patients, whereby the unconsciously self-selected speed for natural walking is a lesser proportion of the potential for maximal safe speed than that used by healthy subjects. Reduced baseline excitability of cortical circuits involved in the preparation and execution of motor command may be involved in producing such a pattern [35–37].

The parameter we defined as *contribution of step length* (CSL) to increased ambulation speed closely estimates the relative contribution of step length changes to changes in speed. It is age-invariant and is easily measured in the clinic. This retrospective study indicates that CSL may provide a useful quantitative marker of disease duration in PD. A long-term prospective, longitudinal study will adequately assess whether this parameter is effectively a marker of disease progression and can be useful to assess the effectiveness of putative disease modifying medications.
